# Glycation of tilapia protein hydrolysate decreases cellular antioxidant activity upon *in vitro* gastrointestinal digestion

**DOI:** 10.1016/j.fochx.2024.101228

**Published:** 2024-02-13

**Authors:** Xiaogang Zhang, Parinya Noisa, Ali Hamzeh, Jirawat Yongsawatdigul

**Affiliations:** a School of Public Health, Zunyi Medical University, Zunyi, Guizhou 563000, China; b School of Food Technology, Institute of Agricultural Technology, Suranaree University of Technology, 111 University Avenue, Nakhon Ratchasima 30000, Thailand; c School of Biotechnology, Institute of Agricultural Technology, Suranaree University of Technology, 111 University Avenue, Nakhon Ratchasima 30000, Thailand

**Keywords:** Maillard reaction products (MRPs), Reducing power, Gastrointestinal (GI) digestion, Fourier-transformed infrared (FTIR) spectroscopy, Cellular antioxidant activity (CAA)

## Abstract

Changes in structural characteristics and antioxidant activity of tilapia hydrolysate glycated with glucose, fructose, or xylose at 90 °C for 12 h, and following *in vitro* gastrointestinal (GI) digestion were investigated. Fourier-transformed infrared (FTIR) band between 1,800 and 1,400 cm^−1^ confirmed the structural modifications of hydrolysate under glycations. Glycation drastically increased ${\text{ATBS}}^{\cdot +}$ and ${\text{ONOO}}^{-}$ scavenging activities (*p* < 0.05) as well as ferric-reducing antioxidant power (FRAP). Xylose was the most effective sugar for glycation, yielding the highest chemical antioxidant activities (*p* < 0.05). However, glycated hydrolysates exhibited lower cellular antioxidant activity (CAA) on HepG2 cell when compared to hydrolysates. The extensive glycation of hydrolysates resulted in lower GI digestibility as confirmed by the FTIR spectra of C=O, C—N, N—H, C—C, C—O, and C—H stretching vibrations. Glycation of tilapia hydrolysates only improved chemical antioxidant activities, but alleviated CAA, especially upon simulated GI digestion.

## Highlights

• Glycation improved chemical antioxidant activities of tilapia hydrolysate.
• The *in vitro* digestibility decreased under excessive glycation with xylose.
• FRAP of glycated hydrolysates reduced upon *in vitro* GI digestion.
• Xylose-glycated hydrolysate had the lowest cellular antioxidant activity (CAA).
• CAA of glycated hydrolysates further reduced upon GI digestion.

## 1 Introduction

Glycation between primary amino and active carbonyl groups is spontaneously initiated during thermal processing, food storage, and physiological conditions (D’Cunha et al., 2022, Nooshkam et al., 2019). Several studies have reported that glycation improved the chemical antioxidant activity of proteins, peptides, and amino acids (Han et al., 2018, Nooshkam et al., 2019). The further extension of glycation to form Maillard reaction products (MRPs) have shown to inhibit lipid oxidation (Nooshkam et al., 2019). Moreover, Maillard browning reaction is considered as one of important strategies to improve sensory characteristics of protein hydrolysate, particularly bitterness and fishy note. However, the antioxidant activity of MRPs and the efficiency of glycation to improve the antioxidant activity of peptides in biological systems remain inconclusive. MRPs from the unicorn leatherjacket skin hydrolysate–galactose system was effective in mitigating the hydrogen peroxide (H_2_O_2_)-induced oxidative stress in human histiocytic lymphoma cells (U937 cells) and DNA damage (Karnjanapratum, O'Callaghan, Benjakul, & O'Brien, 2016). MRPs induced from soybean peptides, *l*-cysteine, and xylose have also retarded galactose-induced aging in Institute of Cancer Research mice by increasing the activities of antioxidant enzymes and total antioxidant activities of serum (He et al., 2019). MRPs from scallop mantle hydrolysates with ribose promoted the cellular antioxidant activity (CAA) to a greater extent than the original hydrolysates (Han et al., 2018). In contrast, several studies have indicated that the accumulation of advanced glycation end products (AGEs), which are part of MRPs, formed endogenously under physiological conditions or consumed through diet is associated with diabetes, cardiovascular diseases, and kidney injury with the underlying mechanisms of intracellular oxidative stress enhancement (D’Cunha et al., 2022, Yan et al., 1994). MRPs from silver carp peptide-glucose caused oxidative stress in diabetic mice (Yao, Han, Dong, Zeng, & Liu, 2016). MRPs obtained from half-fin anchovy hydrolysates and glucose also showed antibacterial activity via inducing extracellular and intracellular H_2_O_2_ production in *Escherichia coli* (Wang, Wei, & Song, 2019). Such discrepancies are attributed to varied MRPs from various reaction systems and different evaluation models, which leads to controversial efficacy of MRPs in biological systems.

The gastrointestinal (GI) digestion of glycated peptides or proteins is also conflicting, as some researchers observed the breakdown of such compounds (Nooshkam & Madadlou, 2016), whereas some observed resistance to digestive enzymes (Yang et al., 2021). Moreover, the antioxidant activities of glycated peptides would likely be varied upon digestion. The chemical antioxidant activities, including *α*, *α*-diphenyl-*β*-picrylhydrazyl free radical (DPPH•) and/or 2,2′-azino-bis(3-ethylbenzothiazoline-6-sulfonic acid) diammonium salt radical cation $\left({\text{ATBS}}^{\cdot +}\right)$ scavenging capacity and ferric-reducing antioxidant power (FRAP) of some MRPs decreased following GI digestion (Chen et al., 2019, Karnjanapratum et al., 2016), whereas ${\text{ATBS}}^{\cdot +}$ and hydroxyl radical (•OH) scavenging capacities of some MRPs did not change upon GI digestion (Chen, Fang, & Wang, 2020). Oxidative stress suppression was observed when the alcohol-induced liver damage mice was fed with MRPs formed by fish scales peptides and xylose (Chen et al., 2020); however, MRPs from silver carp peptide-glucose caused oxidative stress in diabetic mice (Yao et al., 2016). To understand health benefits of protein hydrolysate and its glycated products, the antioxidant activities upon GI digestion should be considered to reflect the more biologically relevant properties. The finding would lay an important groundwork for developing protein hydrolysate and/or its glycated derivatives as functional foods.

In this study, the efficacy of tilapia hydrolysate glycation with three different types of sugar, including glucose, fructose, and xylose, was systematically investigated with respect to the degree of hydrolysis. Changes in chemical antioxidant activities and CAAs along with their structural characteristics upon *in vitro* GI digestion of MRPs were assessed.

## 2 Materials and methods

Potassium bromide (KBr, spectroscopy grade) was purchased from ACROS Organics™ (Morris Plains, NJ, USA). Nitro blue tetrazolium chloride (NBT), quinine hemisulfate salt monohydrate (99.0 %–101.0 %), and 3, 5-dinitrosalicylic acid (DNS, 98 %) were purchased from Sigma-Aldrich (St. Louis, MO, USA). The preparation of tilapia muscle protein powder and its hydrolysates was performed following the protocol described by Zhang, Noisa, and Yongsawatdigul (2020). Defatted tilapia protein powder was prepared to contain 10 % crude protein (w/v), and then pH was adjusted to pH 8.0 by 0.15 M NaHCO_3_ and hydrolyzed by 5 % Alcalase (based on crude protein, w/w) at 50 °C for 2 and 10 h. Following centrifugation and filtration, supernatants were collected and stored at −80 °C. The 2- and 10-h hydrolysates were referred to as *H*_2_ and *H*_10_, respectively.

### 2.1 Glycation of hydrolysates

For glycation, 150 mM of sugar (glucose, fructose, or xylose) and hydrolysate (*H*_2_ or *H*_10_, based on *α*-amino group content, *l*-Leu equivalents) were mixed in a total volume of 3 mL. The reactions were performed in a 20-mL Pyrex screw cap tube and placed in a 90 °C water bath for 0, 2, 4, 6, 8, 10, and 12 h. The tubes were taken out and cooled down in ice water at respective glycation times. The final volume of glycated hydrolysate was brought to 5 mL and stored at −80 °C for further analyses. The glycated hydrolysate was referred to as *S*_t_*H*_n_, where, *S* stands for the type of sugar, including *G* = glucose, *F* = fructose, and *X*  = xylose; t was the glycation time of 0, 2, 4, 6, 8, 10, and 12 h; and *H*_n_ was the 2- or 10-h hydrolysate. For example, *G*_2_*H*_2_ was the glycated *H*_2_ with glucose for 2 h. *G*_0_*H*_2_ represents the mixture of *H*_2_ and glucose without glycation.

### 2.2 Simulated *in vitro* GI digestion

The simulated *in vitro* GI digestion was performed according to the INFOGEST static method with slight modifications as described by Zhang et al. (2020). In the oral phase, *α*-amylase and lipase were ignored as the glycated samples contained mainly monosaccharides, peptides, and MRPs. *S*_t_*H*_n_-*GID* was used to indicate the GI digesta of individual samples (*S*_t_*H*_n_). The enzyme blank (*E*_0_) during GI digestion was prepared without samples. All results were reported following subtraction of the corresponding enzyme blank.

### 2.3 α-Amino group content

The *α*-amino group content of samples was measured using 2, 4, 6-trinitrobenzenesulfonic acid solution (TNBS) as described by Zhang et al. (2020). Absorbance was measured at 420 nm by GENESYS™ 10S UV–Vis Spectrophotometer (Thermo Scientific™, Rochester, NY, USA). *l*-Leu was used as a standard. Results were expressed as *l*-Leu equivalents.

### 2.4 Reducing sugar

Reducing sugar was detected using the DNS method (Miller, 1959) with slight modifications. The DNS reagent was prepared to contain 0.4 M NaOH, 1.06 M sodium potassium tartrate, and 48 mM DNS. A mixture of 200 μL diluted sample and DNS reagent (1:1) was incubated at 95 °C in a water bath for 15 min and subsequently cooled down in ice water. The mixture was subsequently diluted with 1,000 μL DI water, and the absorbance at 540 nm was measured. The corresponding sugar, including glucose, fructose, or xylose, was employed as the standard for the respective glycation system. The residual concentration of each sugar was reported.

### 2.5 Chemical characteristics of glycated hydrolysates

#### 2.5.1 Fructosamine

Fructosamine (1-amino-1-deoxy-d-fructose) was monitored using the NBT assay according to Martinez-Saez, Fernandez-Gomez, Cai, Uribarri, and Castillo (2019) with slight modifications. NBT at 0.23 mM was prepared weekly in 100 mM sodium carbonate buffer (pH, 10.8) and stored at 4 °C. All samples were diluted 20 times; subsequently, 50 μL of each sample was incubated with 950 μL of NBT solution at 37 °C for 20 min. The DI water was used as a blank. Absorbances at 530 nm were reported.

#### 2.5.2 Absorbances at 294 and 420 nm

To monitor glycation-derived colorless and brown products, all samples were diluted 100 and 10 times for measurement of absorbance at 294 and 420 nm, respectively.

#### 2.5.3 Fluorescent intensity

To prevent fluorescent quenching effects and nonlinear responses, the glycated samples and their digesta were diluted 600 and 200 times, respectively. Fluorescence was measured using a JASCO FP-8300 spectrofluorometer (Tokyo, Japan) at *λ*_Ex/Em_ = 347/415 nm with a 5 nm bandwidth (Morales & Jiménez-Pérez, 2001). Quinine hemisulfate salt, which was dissolved in 0.1 M H_2_SO_4_, was used as a standard, and the results were expressed as quinine hemisulfate equivalents (μM).

#### 2.5.4 Size exclusion chromatography (*SEC*)

The molecular weight (MW) distribution was detected as described by Zhang et al. (2020). A Superdex Peptide 10/300 GL column was equipped with fast protein liquid chromatography (ÄKTA PURE 25, GE Healthcare Bioscience Co., Uppsala, Sweden). Subsequently, 100 μL of the diluted sample was loaded onto the column. The eluted compounds were monitored at 214, 294, and 420 nm.

#### 2.5.5 *Fourier-transformed infrared (*FTIR*) spectroscopy*

KBr pellets were prepared, and FTIR spectra were collected by IR transmission analysis using an FTIR spectrophotometer (Bruker Tensor 27, Karlsruhe, Germany). All spectra were recorded within a range from 4,000 to 400 cm^−1^ with 4 cm^−1^ resolution and 64 scans. The triplicate spectra of each sample were averaged, baseline corrected, normalized, and calculated for the second derivative with 13 smoothing points using the OPUS software version 7.5 (Bruker Optics GmbH, Ettlingen, Germany).

### 2.6 Chemical antioxidant activities

Chemical antioxidant activities were performed as described by Zhang et al. (2020) with slight modifications. For the ${\text{ABTS}}^{\cdot +}$ scavenging capacity, the absorbance of the ${\text{ABTS}}^{\cdot +}$ working solution was adjusted to 0.7 ± 0.01 at 734 nm. All samples were diluted 300 times, and 50 μL of each sample was incubated with 950 μL of the ${\text{ABTS}}^{\cdot +}$ working solution for 15 min. Absorbance was measured within 30 min. The DI water was used as a blank. The ${\text{ABTS}}^{∙\;\text{+}\;}$ scavenging capacity was calculated using the following equation:

(1) $$\%{\text{ABTS}}^{\cdot +}\text{scavenging}\;\text{capacity}=\frac{{\text{A}}_{\text{blank}}-{\text{A}}_{\text{sample}}}{{\text{A}}_{\text{blank}}}\times 100\%$$

where, A_blank_ and A_sample_ referred to the absorbances of the blank and sample, respectively.

For FRAP, all samples were diluted 100 times, and 100 μL of each sample was incubated with 1,000 μL of the FRAP reagent at 37 °C for 10 min in the dark, and the absorbance was measured at 593 nm. DI water was used as the blank.

Peroxynitrite (${\text{ONOO}}^{\text{-}}$) scavenging capacity results were expressed as glutathione equivalents (μmol GSH) based on the final volume of glycation (5 mL) or the *in vitro* GI digestion (25 mL).

### 2.7 CAA

The intracellular ROS scavenging capacity was slightly modified based on Zhang et al. (2020). Human hepatocellular carcinoma (HepG2) cells at 6 × 10^4^ cells/well were cultured on a sterile Corning® 96-well black polystyrene microplate with a flat clear bottom until 100 % confluence. Subsequently, cells were further incubated for 1 h with the sample containing 25 μM 2′7′-dichlorodihydro-fluorescein diacetate (DCFH-DA). Subsequently, the medium was discarded, and cells were washed once with 1 × PBS. A free radical generator, 2,2′-azobis (2-methylpropionamidine) dihydrochloride (AAPH), in 1 × PBS at 600 µM was applied. The fluorescence intensity at *λ*_Ex/Em_ = 485/538 nm was read at 37 °C after 1 h incubation. The cells treated by DCFH-DA only, DCFH-DA followed by APPH (oxidative stress control), and ascorbic acid (positive control) were evaluated along with the samples. Furthermore, cells treated by a sample without AAPH-induced oxidative stress were prepared. Results were expressed as the CAA unit.

### 2.8 Statistical analyses

All tests were performed in three independent replicates and expressed as means ± standard deviations. Comparisons between the two means were performed using independent sample t-tests (*p* = 0.05). When there were more than two treatments, the mean differences were analyzed by analysis of variance using Tukey HSD with p < 0.05 (Statistical Package for the Social Sciences version 23, IBM, Armonk, NY, USA)**.**

## 3 Results and discussion

### 3.1 Chemical characteristics of glycated hydrolysates

**Fig. 1:**
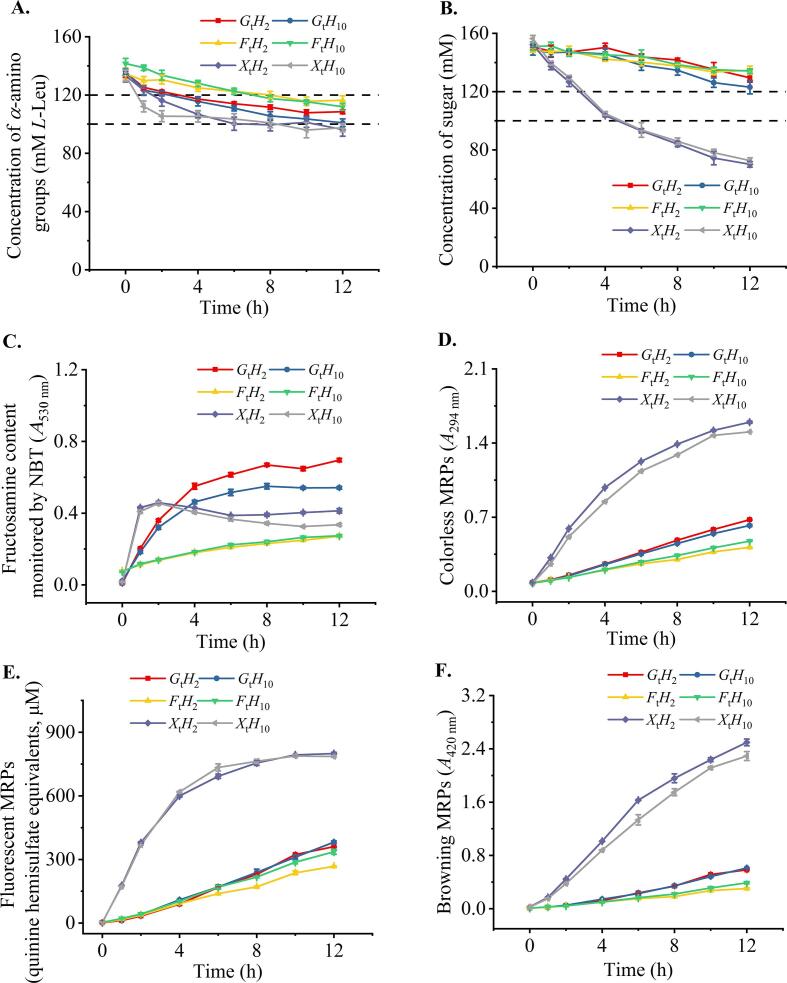
Changes in *α*-amino groups (A), reducing sugars (B), fructosamine (C), colorless Maillard reaction products (MRPs) (D), fluorescent MRPs (E), and browning MRPs (F) during the 12-h glycation of tilapia hydrolysates and various types of sugar. *G* = glucose, *F* = fructose, and *X*  = xylose; t, is the glycation time of 0, 2, 6, and 12 h; *H*_2_ and *H*_10_ are fish hydrolysates prepared from 2- and 10-h hydrolysis, respectively.

The concentration of *α*-amino groups and reducing sugars was reduced with the extended glycation time (*p* < 0.05, Fig. 1A–B). However, fluorescent compounds formed at the early stage of the Maillard browning reaction and advanced MRPs were increased with the extended glycation time (*p* < 0.05, Fig. 1D–F). The Maillard reaction is attributed to the reaction between reducing sugars and *α*-amino group of amino acids and peptides presented in the hydrolysates. Similar trend was observed in glucose-glycated cooked shrimp (*Penaeus vannamei*) hydrolysate system (Nie, Xu, Zhao, & Meng, 2017). Therefore, the significant reduction of xylose (Fig. 1B) indicated that it was the most reactive reducing sugar tested, corresponding to the highest contents of colorless MRP, fluorescent products, and browning index (Fig. 1D–F). Notably, the fructosamine concentration of xylose samples reached the highest at 2 h and slightly decreased thereafter (Fig. 1C), suggesting the rapid formation of early MRPs.

**Fig. 2:**
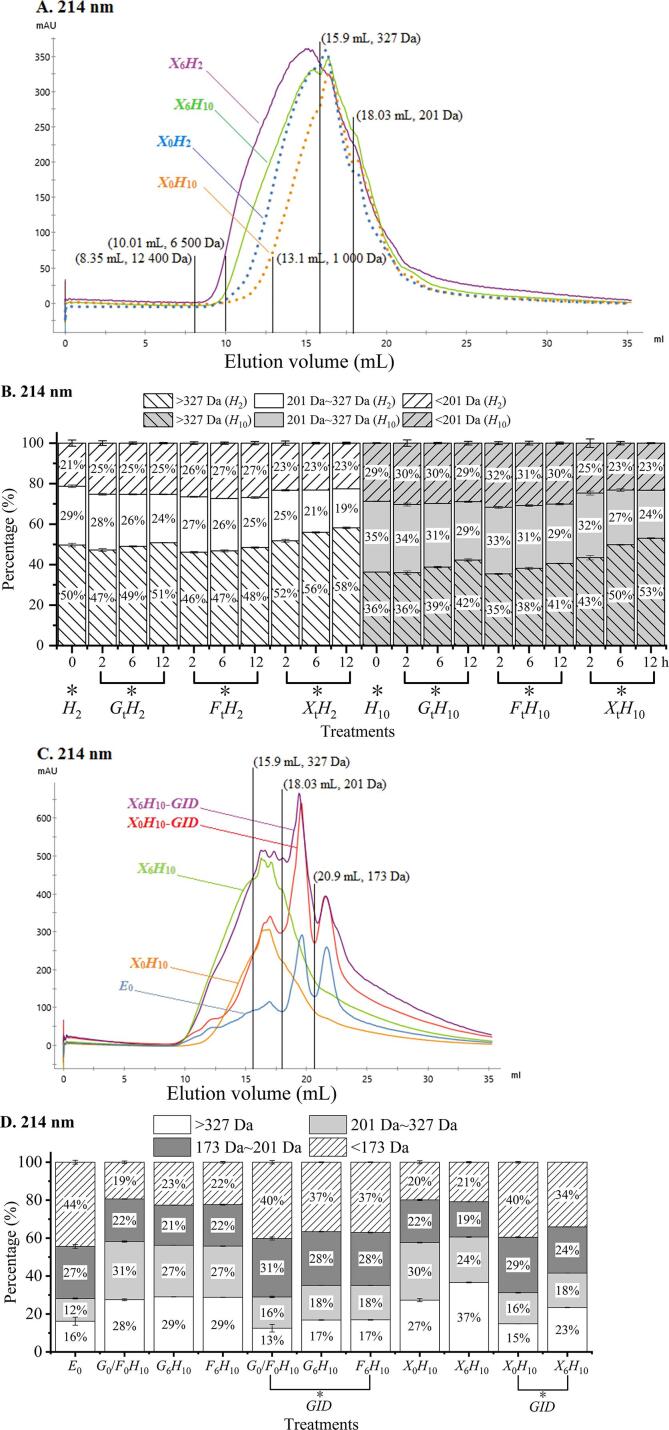
Size exclusion chromatography (*SEC*) monitored at 214 nm and corresponding molecular weight (MW) distribution, the representative chromatogram (A) and MW distribution charts (B) of hydrolysates and glycated hydrolysates, the representative chromatogram (C) and MW distribution charts (D) of hydrolysates and glycated hydrolysates upon gastrointestinal (GI) digestion. MW at elution volume (*V*_e_) is calculated using the following equation: $\log MW=3.7209K_{\mathrm{av}}^{2}-5.6405K_{\mathrm{av}}+4.3691,\;R^{2}=0.9996$. Abbreviations are the same as those described in Fig. 1. The postfix, *GID*, following the sample name is used to indicate the GI digesta of samples. *E*_0_ is the enzyme blank of GI digestion.

The glycation of *H*_2_ and *H*_10_ at equal molarity (*l*-Leu equivalents) showed comparable characteristics regardless of reducing sugars applied (Fig. 1), suggesting that the peptide chain length between *H*_2_ and *H*_10_ was not the main factor affecting the glycation as the majority of peptides **(**>85 %) in both *H*_2_ and *H*_10_ were smaller than 1,000 Da (Fig. 2A–B). Compared with glycine and triglycine, diglycine was the most reactive peptide toward glucose (Kim & Lee, 2009). However, the lower MW peptide fractions from chicken bone peptide showed higher reactivity toward reducing sugars and generated MRPs to a greater extent (Nie et al., 2017).

Xylose, which is an aldopentose, is more reactive than aldohexose (glucose) and ketose (fructose) (Laroque, Inisan, Berger, & Guérard, 2008). This could be associated with the lowest steric hindrance of the accessible carbonyl group of aldopentoses (Laroque et al., 2008, Nooshkam et al., 2019). Furthermore, sugars in open (carbonyl) structure showed higher reactivity than those in the ring (hemiacetal or hemiketal) structure. Xylose is less stable than glucose and fructose, representing a higher proportion of open-chain structure. Furthermore, the interconversion of cyclic furanose of fructose to the acyclic form is slower than that of xylose and glucose, which undergoes via a simple mutarotation (Laroque et al., 2008). Thus, fructose exhibited the lowest reactivity in glycation. The aldehyde carbonyl group of xylose and glucose also possesses higher electrophilicity than that of fructose, thereby leading to their higher reactivity.

### 3.2 MW Distribution of glycated hydrolysates

All hydrolysates and glycated products displayed strong absorption at 214 nm (Fig. 2A); however, hydrolysates were less sensitive at 294 nm and showed no absorption at 420 nm (Fig. S1A'–A''). Similar trend has also been previously observed (Han et al., 2019, Han et al., 2018). Glycated hydrolysates (*X*_6_*H*_2_ and *X*_6_*H*_10_) displayed larger peak areas at 214 nm than individual hydrolysates (*X*_0_*H*_2_ and *X*_0_*H*_10_, Fig. 2A). Therefore, the glycation of hydrolysates generated larger MW species with molar absorptivity at 214 nm.

Peptides in both *H*_2_ and *H*_10_ showed MW < 6,500 Da (Fig. 2A), and the majority of peptides (>85 %) were lower than 1,000 Da. Compared with *H*_10_, *H*_2_ contained larger peptides with MW > 327 Da as smaller peptides were obtained along with prolonged proteolysis (*p* < 0.05, Fig. 2B). Additionally, larger glycated peptides were obtained in *H*_2_ as *S*_t_*H*_2_ contained more compounds with MW > 327 Da than *S*_t_*H*_10_ (*p* < 0.05, Fig. 2A–B). Additionally, the glycation of *H*_2_ (*S*_t_*H*_2_) generated more species of larger intermediate MRPs (monitored at 294 nm) and browning MPRs (monitored at 420 nm) than that of *S*_t_*H*_10_ (Fig. S1A′–B′, A″–B″). All glycated hydrolysates displayed MW < 12,400 Da (Fig. 2A, S1A′, S1A″). The glycation of both *H*_2_ and *H*_10_ generated a higher number of larger molecules (>327 Da), whereas compounds with MW ranging from 327 to 201 Da were reduced as the glycation time increased (*p* < 0.05, Fig. 2B). This indicated that the smaller peptides with the MW of 327–201 Da would be involved in the Maillard reaction to form larger molecules by condensation. Furthermore, larger molecules have been generated through the glycation of scallop female gonad hydrolysates with ribose (Han et al., 2019), wherein molecules with MW > 1,000 Da gradually increased, whereas those with the MW of 200–1,000 Da decreased, and slight changes were observed in molecules with MW < 200 Da. *H*_2_ and *H*_10_ glycated with xylose, *X*_t_*H*_2_ and *X*_t_*H*_10_, showed significant changes in MW distribution (*p* < 0.05), whereas changes in fructose glycation occurred to a lesser extent. Such changes in MW distribution were correlated with the reactivity of sugars as xylose > glucose > fructose. The findings of this study were consistent with the glycation of gelatin hydrolysates from grass carp (*Ctenopharyngodon idellus*) scales (Chen et al., 2019), which reported that ribose was the most effective sugar in glycation, and larger MW species of MRPs were formed compared with those of xylose and glucose.

Intermediate MRPs monitored at 294 nm were observed throughout the elution time (Fig. S1A'), indicating that these products were present in a wide range of MW. Nevertheless, chromatograms at 214 and 294 nm exhibited different profiles, with intermediate MRPs showing a higher proportion of compounds with MW < 201 Da (Fig. S1A'–B') than that monitored at 214 nm (Fig. 2A–B). Moreover, melanoidins were only observed at the initial elution time (Fig. S1A''), suggesting that they are larger MW products (Fig. S1B'').

### 3.3 Chemical antioxidant activity of glycated hydrolysates

**Fig. 3:**
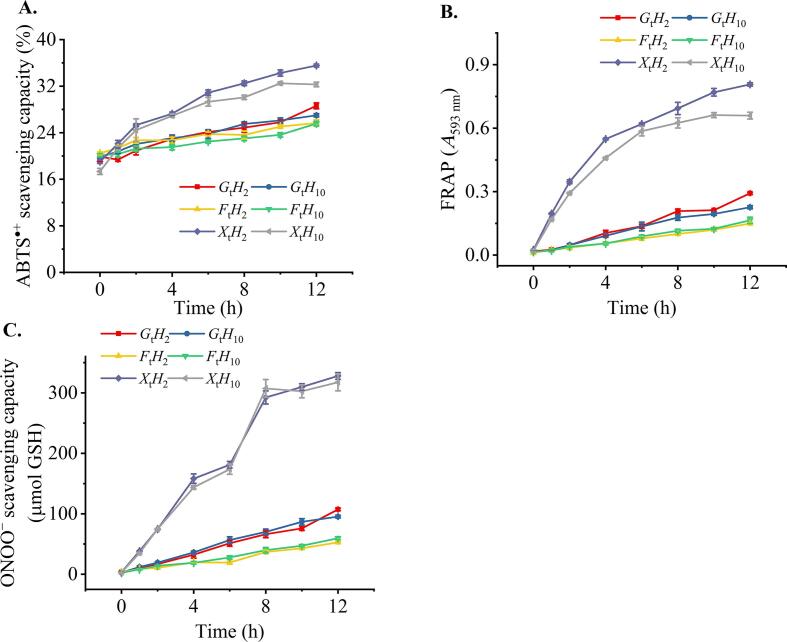
Changes in the chemical antioxidant activities of products during the 12-h glycation, ${\text{ABTS}}^{\cdot +}$ scavenging capacity (A), ferric-reducing antioxidant power (FRAP) (B), and ${\text{ONOO}}^{\;\text{-}\;}$ scavenging capacity (C). Abbreviations are the same as those described in Fig. 1.

Glycation increased chemical antioxidant activities in a time-dependent manner for all samples (*p* < 0.05, Fig. 3), wherein those glycated with xylose (*X*_t_*H*_n_) showed the highest activity. MRPs possessed high reducing power (Fig. 3B), which was consistent with previous studies (Chen et al., 2019). Meanwhile, changes in FRAP and ${\text{ONOO}}^{\text{-}}$ scavenging activities (Fig. 3B–C) were positively correlated with changes in colorless and fluorescent MRPs (Fig. 1D–E), which is in agreement with Kitts (2021) who reported that early stage MRPs with fluorescent characteristics typically exhibited antioxidant activity. However, the extended glycation of the xylose from 8 to 12 h, including *X*_8_*H*_n_, *X*_10_*H*_n_, and *X*_12_*H*_n_, did not show the same rate in increasing antioxidant activities (Fig. 3C) as that of the browning index (Fig. 1F). Browning pigments of melanoidins have been reported to exhibit both pro-oxidant and free-radical scavenging activity (Kitts et al., 2021). These results suggested that the antioxidant activities of MRPs would be mainly attributed from the intermediates of the Maillard reaction, including hydroxyl compounds, heterocyclic pyrrole, and 5-hydroxymethylfurfural (Nooshkam et al., 2019, Zhao et al., 2013). Furthermore, reductone is an intermediate compound, which plays a role in antioxidant activity. However, reductone was rarely generated in acidic conditions (Nooshkam et al., 2019). Thus, it would not be the main product contributing to the antioxidant activity of MRPs in this study.

### 3.4 FTIR

**Fig. 4:**
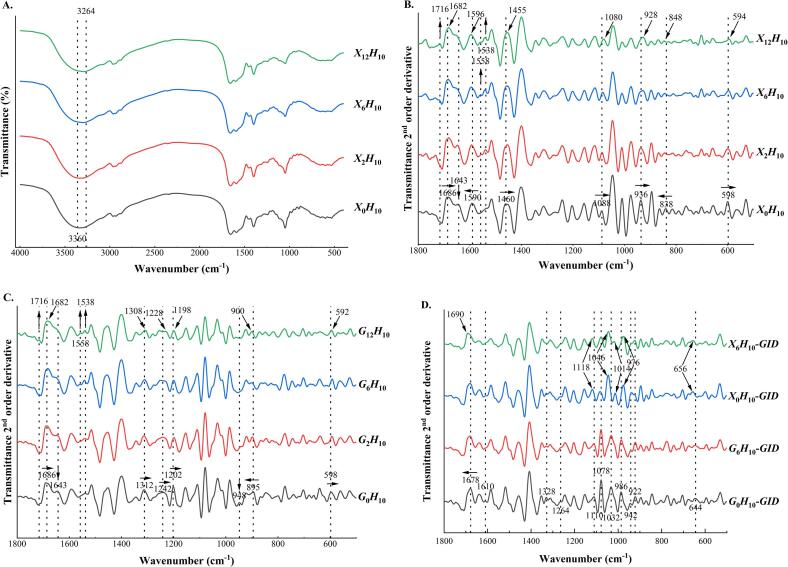
Fourier-transformed infrared (FTIR) spectra (A) and second-order derivative spectra (B–D). *S*_0_*H*_10_, *S*_2_*H*_10_, *S*_6_*H*_10_, and *S*_12_*H*_10_ indicate that the 10-h hydrolysates (*H*_10_) are glycated with sugar (*S*, as *X*  = xylose, *G* = glucose) for 0, 2, 6, and 12 h, respectively. The postfix, *GID*, following the sample name is used to indicate the GI digesta samples. (For interpretation of the references to colour in this figure legend, the reader is referred to the web version of this article.)

Glycated hydrolysates showed similar FTIR spectra as the typical ones of xylose-hydrolysate (Fig. 4A). The absorption band at 3,600–3,200 cm^−1^ has been assigned to the O—H stretching of hydroxyl groups and N—H stretching in the amide A band **(**Mohsin et al., 2018, Oracz and Zyzelewicz, 2019**)**. Band broadening from 3,360 to 3,264 cm^−1^ indicated a disordered structure with an increase in the glycation time (Fig. 4A). The second-order derivatives of spectra showed a distinct peak at 1,716 cm^−1^ in the sample subjected to extended glycation (Fig. 4B). This peak indicated the stretching band of COOH or C=O (Coates, 2000), corresponding to organic acids, aldehydes, melanoidins, and other carbonyl compounds, including the early intermediates, 1-amino-1-deoxy-d-fructose (Amadori compound), and furfural, formed during glycation. Additionally, the direct dehydration of 1-deoxyosone generated carbonyl compounds, including maltol and isomaltol. The IR spectra of melanoidins from glucose and alanine also showed a distinct peak at 1,717 cm^−1^ (Mohsin et al., 2018). The band at 1,695–1,620 cm^−1^ was the C=O stretching of amide I (Foggia, Taddei, Torreggiani, & Tinti, 2012), which was also the overlapping band of the C=O stretching of flavonoids, phenolic acids and its derivatives, quinones, and lipids (Oracz et al., 2019), and the N—H bending vibrations from amine or amide groups (Coates, 2006, Oracz and Zyzelewicz, 2019). The shift from 1,686 to 1,682 and from 1,590 to 1,596 cm^−1^ and the planished band at 1,643 cm^−1^ indicated the structure changes of C=O and N—H during glycation, which would be attributed to products from glycation, including organic acids, carbonyl compounds, and pyrroles. The spectra of melanoidins from glucose and alanine at 160 °C showed a broaden peak at 1,593 cm^−1^ (Mohsin et al., 2018). The asymmetric stretching of carboxylate groups (COO^—^) was approximately 1,594 cm^−1^ (Güler, Vorob'ev, Vogel, & Mäntele, 2016). Therefore, the band shifting from 1,590 to 1,596 cm^−1^ would further suggest the generation of organic acids from glycation. The newly visible band at 1,558 and 1,538 cm^−1^ during glycation would be the mixture of in-plane N—H bending and C—N stretching of amide II (Wubshet et al., 2017) or N—H bending deformation (Siewe, Kudre, Bettadaiah, & Bhaskar, 2020) and the COO^—^ asymmetric stretching at approximately 1,555 cm^−1^ (Rahmelow, Hübner, & Ackermann, 1998). The band shifting from 1,460 to 1,455 cm^−1^ would indicate changes in the C—H bending or deformation vibrations of CH_2_ and CH_3_ of amino acid side chains (Foggia et al., 2012, Mohsin et al., 2018). The shift from 1,088 to 1,080 cm^−1^ corresponded to the C—O—C and C—O stretching vibrations of the glycoside linkage and C—O bond stretching vibration, such as in glycerol (Oracz et al., 2019). Therefore, the prominent changes between 1,800 and 1,400 cm^−1^ indicated the formation of organic acids, aldehydes, melanoidins, and other carbonyl- and nitrogen-containing compounds under glycations.

Bands at 1,400–650 cm^−1^ are associated with the C—O, C—C, and C—N single bond stretches, C—H bending vibrations, and some benzene rings (Batista, de Andrade, Ramos, Dias, & Schwan, 2016). The “saccharide” band at 1,180–953 cm^−1^ results from the stretching of C—C and C—O and the bending mode of C—H bonds (Gu, Kim, Abbas, & Chen, 2010). Oracz et al. (2019) showed that the characteristic absorption bands between 900 and 600 cm^−1^ would be because of the stretching vibrations of the entire anhydroglucose ring. Therefore, bands shifting from 1,088 to 1,080, 936 to 928, 838 to 848, and 598 to 594 cm^−1^ would correlate with a decrease in xylose and an increase in glycoside linkages during glycation (Fig. 4C). Bands at 1,100–600 cm^−1^ of xylose-hydrolysate were different from those of glucose, which would be because of the varied extent of glycations. In the glucose-hydrolysate system, bands at 1,590 and 1,460 cm^−1^ did not shift; however, shifts of 1,312, 1,242, and 1,202 cm^−1^ were observed. Bands at 1,300–1,200 cm^−1^ indicate the C—N stretching and N—H bending motions of the amide Ⅲ band, as well as the overlapping with strong C—O stretching bands and the O—H bending vibration (Foggia et al., 2012, Siewe et al., 2020). The bands of tyrosine sensitive to the hydrogen bond are also located at 1,230–1,270 cm^−1^ (Barth, 2000). The characteristic bands of xylose-hydrolysate glycation were between 1,455 and 1,596 cm^−1^; however, those of glucose-hydrolysate glycation were at 1,300–1,200 cm^−1^.

### 3.5 Characteristics and chemical antioxidant activity of *in vitro* digested glycated hydrolysates

**Fig. 5:**
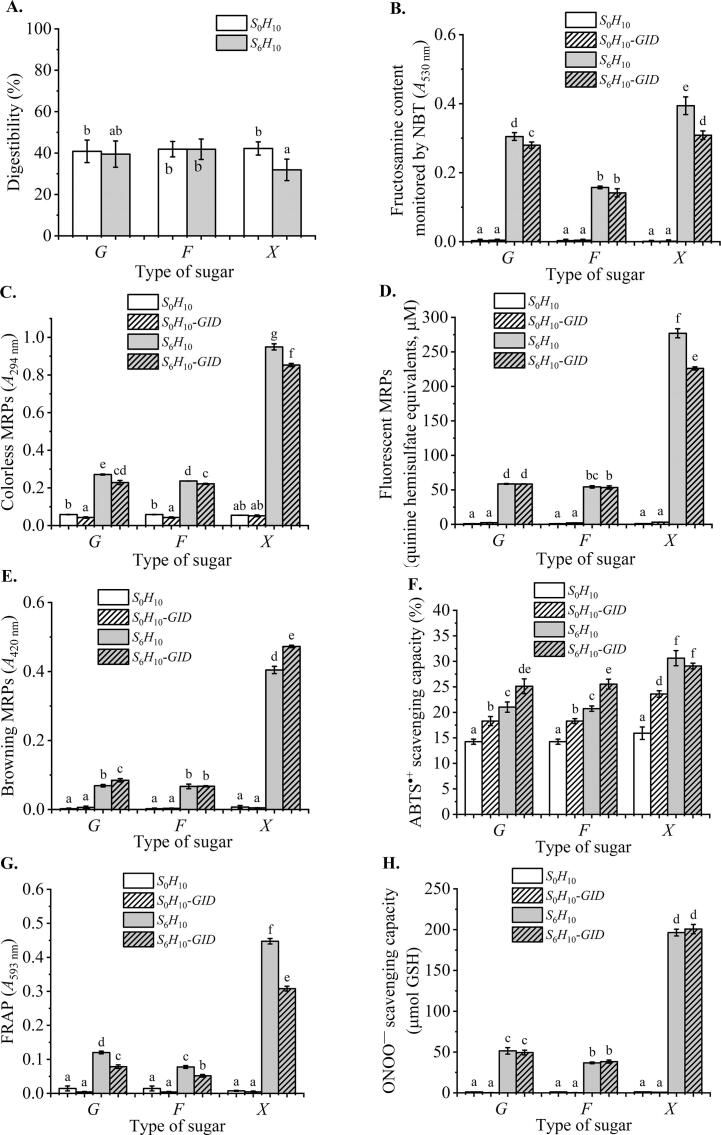
Digestibility, Maillard browning intermediates and products, and the chemical antioxidant activities of glycated hydrolysates upon *in vitro* GI digestion. Digestibility (A), fructosamine (B), colorless MRPs (C), fluorescent MRPs (D), browning MRPs (E), ${\text{ABTS}}^{\cdot +}$ scavenging capacity (F), FRAP (G), and ${\text{ONOO}}^{-}$ scavenging capacity (H). Abbreviations are the same as those described in Fig. 2. *S* indicates various types of sugar as *G* = glucose, *F* = fructose, and *X*  = xylose. Different lowercases indicate the differences in mean values within various treatments (*p* < 0.05).

The digestibility of the hydrolysate (*S*_0_*H*_10_) and *F*_6_*H*_10_ were comparable (*p* > 0.05), whereas that of *X*_6_*H*_10_ was the lowest (*p* < 0.05) (Fig. 5A). Digestibility was negatively correlated to the extent of glycation (Fig. 1). Higher degree of glycation resulted in a lesser susceptibility to digestive enzymes which can occur due to various mechanisms regarding peptic and gastric phases. It has been reported that steric hindrance caused by structural modifications within glycation could retard pepsin accessibility. Lysine and arginine residues as the cleavage sites of trypsin and carboxypeptidase B in gastric phase can be occupied by MRPs (Deng, Wierenga, Schols, Sforza, & Gruppen, 2017; Whitcomb & Lowe, 2007). Such a resistance to digestion has been observed in glycated casein, β-lactoglobulin and ovalbumin among others (Corzo-Martínez et al., 2010, Lugt et al., 2020; Yang et al., 2021), in which lysine content was not significantly changed in glycated sample during digestion by trypsin, while it increased in un-glycated ovalbumin due to trypsin digestion. However, Deng et al. (2017) reported that *α*-chymotrypsin did not hydrolyze the glycated sites of lysine/arginine of *α*-lactalbumin. Additionally, adding sugar to the *N*-terminus of peptides reduced the accessibility of aminopeptidases (McClellan & Garner, 1980).

The contents of fructosamine, colorless MRPs, and fluorescent adducts decreased upon the GI digestion of glycated hydrolysates (*p* < 0.05, Fig. 5 B–D), whereas that of browning MRPs increased (*p* < 0.05, Fig. 5E). It was likely that more advanced glycated products were formed from intermediates generated from GI digestion. Lugt, Venema, Leeuwen, & Bast (2020) reported that the arginine-derived methylglyoxalderived-hydroimidazolone-1 in the GI tract increased to more than 400 %. Smaller compounds were also formed upon the *in vitro* GI digestion of glycated hydrolysates, corresponding to the shift in FTIR bands from 1,678 to 1,690 cm^−1^ in hydrolysate digesta of *X*_6_*H*_10_-*GID* (Fig. 4D). Small bands at 1,610, 1,328, 1,264, and 942 cm^−1^ were observed in *X*_0_*H*_10_-*GID*; however, they decreased in *X*_6_*H*_10_-*GID*. The band at 922 cm^−1^ became more distinct in *X*_6_*H*_10_-*GID* than in *X*_0_*H*_10_-*GID*. Flavonoids, phenolic acids, and quinones can also be observed from FTIR spectra at 1,695–1,630 cm^−1^ (Oracz et al., 2019). The N—H bending vibrations of the amide I band, the C—N stretching vibrations of the amide Ⅲ band, and the O—H bending vibrations were assigned at 1,650–1,590, 1,360–1,310, and 1,410–1,220 cm^−1^, respectively (Coates, 2000). The C—O, C—C, and C—N single bond stretches, C—H bending vibrations, and some bands of benzene rings were at 1,400–650 cm^−1^ (Batista et al., 2016). These FTIR results suggested the newly formed hydroxyl, carbonyl, or phenolic compounds during glycation, and the highly polymerized melanoidins were indigestible as the functional groups of C—N, N—H, C—C, C—O, and C—H restricting vibrations still existed in the digesta.

The ${\text{ABTS}}^{\cdot +}$ scavenging capacity of the digesta of glucose- and fructose-glycated hydrolysates were higher than that of respective hydrolysates and glycated hydrolysates (Fig. 5F). In contrast, the *X*_6_*H*_10_-*GID* activity was comparable with that of *X*_6_*H*_10_. The lowest digestibility of the xylose-glycated hydrolysate (*X*_6_*H*_10_) implies the least number of peptides/amino acids formed upon *in vitro* GI digestion (Fig. 5A), thereby resulting in the subtle changes in the ${\text{ABTS}}^{\cdot +}$ scavenging capacity. All hydrolysates and their digesta showed negligible reducing power (Fig. 5G–H). Glycation significantly increased the reducing power of hydrolysates (Fig. 3B–C). However, GI digestion significantly decreased the FRAP value of all glycated hydrolysates (*p* < 0.05, Fig. 5G). Similar trend was also observed in fructosamine, colorless MRPs, and fluorescent adducts following the GI digestion of glycated hydrolysates (Fig. 5B–D). GI digestion did not affect the ONOO^–^ scavenging capacity of all glycated hydrolysates (*p* > 0.05, Fig. 5H).

It can be concluded that highly glycated hydrolysates had a negative effect on peptide digestibility through structural modifications and the generation of larger MW compounds, wherein the glycated peptides were less susceptible to digestive enzymes. GI digestion decreased the ferric-reducing power of all glycated hydrolysates; however, it had no effect on the ONOO^–^scavenging capacity. The ${\text{ABTS}}^{\cdot +}$ scavenging capacity increased upon *in vitro* GI digestion in the less intense glycated hydrolysate system of glucose and fructose.

### 3.6 CAA

**Fig. 6:**
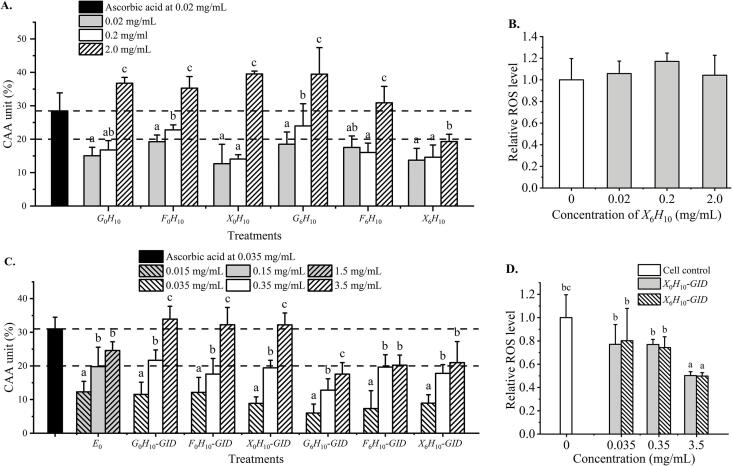
Intracellular ROS scavenging capacity (A) and pro-oxidation (B) of hydrolysates and glycated hydrolysates, intracellular ROS scavenging capacity (C) and pro-oxidation (D) of the digesta of hydrolysates and glycated hydrolysates. Abbreviations are the same as those described in Fig. 2. *E*_0_ is the enzyme blank of GI digestion. Different lowercases indicate the differences in the mean values of samples (*p* < 0.05).

All glycated hydrolysates and their digesta were non-toxic to HepG_2_ cells up to 3.5 mg/mL. The intracellular ROS scavenging capacity of all samples was concentration-dependent, wherein hydrolysates at 10 h, fructose- and glucose-glycated hydrolysates, namely *S*_0_*H*_10_, *F*_6_*H*_10_, and *G*_6_*H*_10_, showed comparable CAA at the same concentrations (*p* > 0.05, Fig. 6A) despite higher chemical antioxidant activities of glycated hydrolysates (Fig. 3). Meanwhile, the xylose-glycated hydrolysate (*X*_6_*H*_10_), which has the greatest extent of glycation and the highest chemical antioxidant activities, showed the lowest CAA (*p* < 0.05, Fig. 6A). It has been reported that glycated proteins induced cellular oxidative stress (Yan et al., 1994) and caused cytotoxicity to BHK 21 hamster fibroblast cells and SHSY5Y human neuroblastoma cells (Loske et al., 1998). Dihydropyrazines formed by dimerization of glucosamine or 5-aminolevulinic acid have been reported to induce oxidative stress in HeLa cell (Miyauchi, Koba, Sawai, Kansui and Takechi, 2023). However, in this study, the *X*_6_*H*_10_ at approximately 0.02–2.0 mg/mL did not show the cellular pro-oxidative effect (Fig. 6B, *p* > 0.05). The lower antioxidant activity of *X*_6_*H*_10_ may be partly attributed to larger MW MRPs, including melanoidins, formed in this most reactive glycation. Kitts, Chen, & Jing (2012) reported that low MW MRPs were more effective than their high MW counterparts in protecting Caco-2 cells against oxidation. Lower MW MRPs were easily more absorbed into intracellular compartments (Poulsen et al., 2013). Thus, the lower MW of melanoidins and the higher proportion of un-glycated peptides in *F*_6_*H*_10_, and *G*_6_*H*_10_ would contribute to higher CAA than that of *X*_6_*H*_10_ (Fig. 6A), which contained the highest MRPs (Fig. 1E-F). It should be mentioned that glycated hydrolysates did not show higher CAA than that of respective hydrolysate counterparts despite of their higher chemical antioxidant activities. Cellular absorption of glycated proteins and MRPs are likely different from that of peptides, which could partly contribute to such discrepancy. Zhao et al. (2019) reported that free AGEs are weakly absorbed, mostly through simple diffusion, which is not an effective transport pathway. While peptide transporters 1 (PEPT1) pathway can mainly be involved in absorption of peptide AGEs. Since glycated peptides are resistant to hydrolysis of digestive enzymes, their insufficient degradation results in lower absorption (Yuan et al., 2023, Zhao et al., 2019). In addition, binding of AGEs to type I cell surface receptor (RAGE) has been reported to increase cellular reactive oxygen species (ROS) production and inflammatory response (Ott et al., 2014). Lower cellular uptake and increased ROS would partly explain lower CAA observed in glycated tilapia hydrolysate samples.

It should be noted that peptides from the enzyme blank (*E*_0_) of GI digestion also showed a significant CAA in a concentration-dependent manner (Fig. 6C), suggesting the contribution of antioxidant activities from digestive enzymes. This should be taken into consideration when evaluating CAA of sample digesta. All the digesta of glycated hydrolysates showed lower CAA than the respective hydrolysate digesta (Fig. 6C). The *X*_6_*H*_10_-*GID* contained more and larger MW compounds than *G*_6_*H*_10_-*GID* and *F*_6_*H*_10_-*GID* (Fig. 2D). The state of AGEs after GI digestion led to the various absorption mechanisms and cellular activities. Larger MW compounds would hamper cellular absorption (Kitts et al., 2012, Poulsen et al., 2013), which could be observed in *X*_6_*H*_10_ and *X*_6_*H*_10_-*GID* with the highest degree of browning in concomitant with the lowest CAA.

The digesta of hydrolysate and xylose-glycated hydrolysate did not show pro-oxidation to Hep-G2 cells (Fig. 6D). Chemical and CAA of hydrolysate and glycated hydrolysates are modified upon GI digestion (Fig. 5, Fig. 6). Glycation improved the chemical antioxidant activities of peptides but reduced their intracellular antioxidant activities. The underlying mechanisms of MRPs absorption, quantitative structure–activity relationship (QSAR) and the absorption interaction between MRPs and peptides need further investigation.

## 4 Conclusion

Glycation of hydrolysate was significantly intensified with the extended reaction time of 12 h at 90 °C and xylose was the most reactive sugar with approximately 2.3-time higher in fluorescent MRPs than glucose and fructose. Glycation improved the chemical antioxidant activity, particularly the reducing power, which was correlated with the intermediates of the Maillard reaction including hydroxyl compounds, heterocyclic pyrrole, and 5-hydroxymethylfufural. Hydrolysates with extended glycation showed lower digestibility, implying lower bioavailability. The *in vitro* GI digestion decreased the chemical antioxidant activities of glycated hydrolysates, particularly the highly glycated samples. Furthermore, the glycated hydrolysates and their digesta showed lower intracellular ROS scavenging activity than those of respective hydrolysates. Structural modifications through glycation process and gastrointestinal digestion led to a reduction of cellular antioxidant activity. The glycation of tilapia hydrolysates would be effective for improving its chemical antioxidant activity for food applications, but not effective for improving the antioxidant activity in the biological system. Further *in vivo* studies of glycated tilapia hydrolysate, particularly absorption and health benefits, would pave the way to functional food development of glycated tilapia protein hydrolysate.

## CRediT authorship contribution statement

**Xiaogang Zhang:** Formal analysis, Investigation, Methodology, Writing – original draft, Writing – review & editing, Validation, Visualization. **Parinya Noisa:** Formal analysis, Methodology, Supervision. **Ali Hamzeh:** Formal analysis, Methodology, Writing – review & editing. **Jirawat Yongsawatdigul:** .

## Declaration of competing interest

The authors declare that they have no known competing financial interests or personal relationships that could have appeared to influence the work reported in this paper.

## Data Availability

Data will be made available on request.
