# Supplementary material for: Glycation of tilapia protein hydrolysate decreases cellular antioxidant activity upon in vitro gastrointestinal digestion
Source: Food Chem X. 2024 Feb 13;21:101228. doi: 10.1016/j.fochx.2024.101228 (PMC10881548; doi:10.1016/j.fochx.2024.101228)
Supplement: Supplementary Data 1 [file mmc1.docx]

**Supplementary**

**Fig. S1** Size exclusion chromatography (*SEC*) and corresponding MW distribution. The typical chromatograms at 294 nm (A′) and 420 nm (A″) and MW distribution charts of glycated hydrolysate (B′ & B″), the typical chromatograms at 294 nm (C′) and 420 nm (C″) and MW distribution charts of digesta of glycated hydrolysate (D′ & D″). MW at elution volume (*V*_e_) was calculated using the equation of $\log\text{MW}\text{=3.7209}\text{K}_{\text{av}}^{\text{2}}\text{-5.6405}\text{K}_{\text{av}}\text{+4.3691, }\text{R}^{\text{2}}\text{=0.9996}$, and used to determine of MW of compounds. *G* = glucose, *F* = fructose, and *X* = xylose; the footnote, t, was the glycation time of 0, 2, 6, and 12 h; *H*_2_ and *H*_10_ were fish hydrolysate prepared from 2 and 10 h hydrolysis, respectively. The postfix, *GID*, following the sample name was used to indicate the GI digesta of samples. *E*_0_ was the enzyme blank of GI digestion.
